# An Ecological Approach to Conceptual Thinking in Material Engagement

**DOI:** 10.5964/ejop.13227

**Published:** 2024-05-29

**Authors:** Nicolás Alessandroni, Lambros Malafouris, Shaun Gallagher

**Affiliations:** 1 Concordia Infant Research Laboratory, Concordia University, Montréal, Canada; 2 Hertford College/Institute of Archaeology, University of Oxford, Oxford, United Kingdom; 3 Department of Philosophy, University of Memphis, Memphis, TN, USA; 4 School of Liberal Arts, University of Wollongong, Wollongong, Australia; Tampere University, Tampere, Finnland

**Keywords:** material engagement theory, conceptual thinking, things, material culture, ecological-enactive cognition

## Abstract

Although post-cognitivist approaches have shaken the status quo by emphasising the dynamic interactions among the brain, the body, and the environment in cognition, mainstream psychological theories continue to view concepts as primarily representational or skull-bound mental phenomena. As a result, the dynamics of action and the possible impact of material culture on conceptual thinking are poorly understood. In this paper, we explore the process and meaning of conceptual thinking from a material engagement perspective. We argue that conceptual thinking is not a matter of forming representations in the head but something we do—a way of engaging with materiality. Conceptual thinking is conceptual thinging, namely a kind of unmediated practical knowledge that individuals put into play when they engage, in a general way, with and through the world. In this sense, we propose that conceptual thinking is instantiated in the dynamic coordination of bodily practices and artefacts in sociomaterial activities. To elucidate this perspective, we introduce seven principles defining conceptual thinking within an ecological-enactive framework of cognition.

Categorisation and conceptual thinking are pivotal in cognitive research, regarded as the building blocks of cognition by many scholars (e.g., Harnad, 2005). Without concepts, thinking in general terms would be impossible, potentially leading to experiencing the world as a perpetual series of distinct entities or facts, thus hindering the development of critical skills such as memory, learning, action planning, self-regulation, and communication (Oakes & Rakison, 2003; Shanks, 2015).

Prevailing theories in philosophy and cognitive science have characterised concepts as representational and brain-bound entities.^1^1For example, Margolis and Laurence (2007) call this the *psychological view* of concepts: “Concepts are mental representations. They are the constituents of propositional attitudes such as beliefs and desires. The psychological view is the default position in many areas of cognitive science and enjoys a good deal of support in the philosophy of mind. It is at the centre of a rich and powerful model of the mind (…)” (p. 563); see also Fodor (1987, 1998). Although acknowledging possible differences in the ontology, internal structure, and relationship of concepts with the external world, theories have indeed construed conceptual thinking as one of many *representation-hungry domains of cognition* (Clark & Toribio, 1994). However, in recent years, contemporary post-cognitivist approaches (e.g., embodied, embedded, enactive, extended, and ecological frameworks) have shaken the status quo in this regard.

By focusing on the dynamic interactions between the brain, body, and environment rather than on internal information processing (Gallagher, 2017; Newen et al., 2018), contemporary perspectives redefine cognition as *adaptive behaviour*, which refers to “the sum of all flexible, skillful capacities that an organism possesses for dealing with the environment” (Heras-Escribano, 2021, p. 338). From such a viewpoint, various scholars have convincingly offered non-representationalist explanations of cognitive phenomena, including motor skills and their development (Adolph, 2019; Travieso et al., 2020), situated anticipation (van Dijk & Rietveld, 2021), action planning (Keen et al., 2014; Ossmy et al., 2020), creativity and innovative action (Baber et al., 2019; Malafouris, 2014; Yakhlef & Rietveld, 2020), tool making and tool using (Malafouris, 2018, 2021; Nonaka & Stoffregen, 2020; Overmann & Wynn, 2019), conversation and linguistic thought (Di Paolo et al., 2018; Gallagher, 2020a; Kiverstein & Rietveld, 2021), memory (Prezioso & Alessandroni, 2023; Sutton et al., 2010), and social cognition (Abramova & Slors, 2015; De Jaegher et al., 2017; Lindblom, 2020).

Post-cognitivism is rapidly moving towards a *sociomaterial practice-based explanation* of cognitive phenomena (Malafouris, 2018, 2020). For instance, the Skilled Intentionality Framework (Rietveld et al., 2018; Rietveld & Kiverstein, 2014) proposed to analyse cognition in terms of selective engagement with multiple affordances, achieved through participation in sociomaterial normative practices (see also Heras-Escribano & De Pinedo-García, 2018, for an analysis based on Niche Construction Theory). Likewise, building upon the research of anthropologist Charles Goodwin (2000, 2002, 2013), Gallagher (2020a) argued that within a conversation “meaning emerges at the intersection of a set of semiotic resources that include social, cultural, material structures and their dynamical changes in the environment where action and interaction occur” (p. 59). Nonetheless, even with the notable advancements within post-cognitivist paradigms, there remains a need for a comprehensive understanding of conceptual thinking and other psychological processes that have conventionally been viewed as detached from real-world interactions (Kiverstein & Rietveld, 2018; Shapiro 2011). This challenge is commonly referred to as the *scaling-up problem* (Chemero, 2009; Petracca, 2023).

This paper seeks to reframe the characterisation of conceptual thinking by considering both the enactive contexts and the processes of material engagement that underpin it in everyday life. While not attempting to provide a complete theory of conceptual thinking, we aim to develop a programmatic statement on how conceptual thinking could be re-envisioned through the insights offered by Material Engagement Theory (MET) (Malafouris, 2013, 2019, 2020). We begin by providing a concise overview of mainstream psychological perspectives on concepts and identifying two major problems with these theories: (i) treating concepts as detached from action dynamics, and (ii) neglecting the constitutive role of material culture in cognition. We then introduce the fundamentals of Material Engagement Theory (MET) and propose seven principles to define conceptual thinking, integrating enactive, ecological, extended, distributed, and semiotic elements. We argue that conceptual thinking is not the entertaining of representations in the head but a way of engaging with materiality: a kind of generalised and practical knowledge that individuals put into play when they engage *with* the world. In this regard, we propose that concepts arise from the dynamic coordination between bodily practices and artefacts in sociomaterial activities and are distributed in the brain-body-world networks from which they emerge.

## Mainstream Concept Theories and Their Problems

The classical theory of concepts, which can be traced back to the philosophical contributions of Aristotle, posits that concepts are abstract definitions of a class that are stored in the mind as representations (Cohen & Murphy, 1984; Lakoff, 1990). This theory also asserts that concepts have two main components: an intensional core and an extensional core. The intensional core refers to the set of sufficient and necessary conditions that an entity must meet to be considered an exemplar of the category the concept represents. The extensional core, on the other hand, refers to the complete set of entities that can be considered instances of the concept (Murphy, 2002).

Because of their allegedly propositional nature, concepts have been traditionally defined as language-like or logical entities. For instance, Brown (1956/2009) argued that acquiring a concept is equivalent to matching environmental groupings with names. Similarly, Archer (1966) proposed that concepts are linguistic *labels* attached to the world. From a more radical perspective, Vygotsky (1934/2008) asserted that “real concepts are impossible without words, and conceptual thought does not exist beyond verbal thought” (p. 115); Lamb (1970) posited that a concept equals the meaning of a word; and Whorf believed that language “shapes the way we think, and determines what we can think about” (Whorf, 1956). Similarly, other authors assume the existence of a natural pairing between concepts and formal logical abilities. For instance, Piaget maintained that preverbal infants lack even “preconcepts,” and claimed that “true concepts” develop no sooner than the *concrete operational stage*, typically spanning the ages of 7–11 years (Piaget, 1951/1999).

In the last 50 years, the “concepts-as-definitions” account has faced challenges from both theoretical and empirical research. Critiques primarily concern the internal structure of concepts (i.e., their components and the relations between them) and the organisation of the human conceptual system (i.e., relations between concepts). For instance, the *prototype theory* proposed that conceptual representations do not encode sufficient and necessary conditions, but rather a statistical analysis of the properties that category members tend to have (Mervis & Rosch, 1981; Rosch, 1975). In this view, concepts are organised around prototypes, which are average category members presenting the most common attribute configurations. These prototypes are summary abstractions that do not necessarily correspond to real objects in the world (Rosch, 1998), but are statistical benchmarks that help determine the degree to which a given entity falls within a category and how good an exemplar it is (e.g., a dining-room chair is a better example of the chair concept than a dentist’s chair, and a table is a better example of the furniture concept than a clock). By referring to the problem of categorical membership as a matter of degree, the prototype theory introduced the idea that family resemblance, rather than criterial features, better explains how categories are bounded (Rosch & Mervis, 1975).

In contrast, the *exemplar theory* denied the existence of unique and unitary descriptions for all class members. Instead of seeing concepts as summary representations, this theory regards them as *sets of exemplars* available in the memory that can be retrieved upon environmental requirements (Smith & Medin, 1981). In other words, the table concept is equivalent to the set of tables a subject remembers, and to have the table concept is to remember table instances (Medin & Schaffer, 1978).

The prototype and exemplar theories proposed alternative cognitive architectures to explain the internal organisation of concepts, yet their divergence from traditional theory is not as substantial as it may seem. Despite differences in what concepts represent (i.e., definitions, prototypes, or concrete exemplars), all theories agree that conceptual thinking is a representational affair. Furthermore, all theories assume a clear ontological distinction between the subject categorising and the categorised world. This suggests that concepts are useful in enabling subjects to map the mind-independent ontological structure of the world for cognitive manipulation (Laurence & Margolis, 1999). To grasp how concepts, as mental contents, establish a connection with the world, cognitive research has provided the standard explanation that this capacity arises from its roots in perception. Indeed, mainstream concept theories inherited the longstanding empiricist view which takes perception to be the “direct, immediate awareness of discriminated existents which results from patterns of energy absorption by groups of receptors” (Efron, 1969, p. 147). In this view, subjects get to know the world “as it is” (see Le Morvan, 2004) thanks to the information they receive through their senses and build concepts through the recognition, correlation, and abstraction of similar perceptual information (e.g., Cahen & Tacca, 2013; Eimas & Quinn, 1994; Horst et al., 2005; Kagan, 1966; Prinz, 2002; Younger, 2003; Zentall et al., 2018).^2^2Note that the idea that concepts are perceptually based has not prevented empiricists from accounting for abstract concepts such as democracy or morality. For a discussion, see Prinz (2002, 2005). As concepts develop from percepts, it is often assumed that they carry in themselves a natural connection with the world (for a critique, see Alessandroni & Rodríguez, 2019).

Although widely accepted, the views of conceptual thinking as abstract representations of the external world, and perception as the reception of information are not without their challenges. For example, these views suggest that conceptual meaning is independent of the subject's categorisation activity, with perception serving as a means to extract information and concepts serving as codifications of that information in our minds. At this point, it would be sensible to ask if there is any relationship between concept formation and the actual engagement of the subject *with* the world in the context of sociomaterial activities. We argue that such a relationship exists and is, in fact, constitutive. In the upcoming paragraphs, we delve into two issues with conventional theories to support this conclusion.

### Perception Cannot Do Without Action

Mainstream theories contend that concepts are formed by integrating similar *sensory atoms* (see Orlandi, 2019). From this perspective, a significant challenge is explaining how individuals select specific stimuli from the virtually infinite array the world offers, as objects themselves lack inherent instructions about their relevance. Consequently, it becomes necessary to resort to some external criterion to categorically parse the environment (Gelaes & Thibaut, 2004). Tomasello sums up this issue very well when he states that:

The problem is that any given set of things has innumerably many similarities, and the concept-forming individual encounters these exemplars one at a time over time without any guidance about what to pay attention to (Tomasello, 2002, p. 6).

Certainly, finding an external criterion becomes elusive when perception is considered an act of contemplation and individuals are viewed as passive observers of environmental spectacles (see Kelly, 2007).

Authors in the traditions of pragmatism, phenomenology, and ecological-enactive theories of cognition have proposed ways to overcome this dilemma. Recent work in this area has sometimes been called a ‘pragmatic turn’ in cognitive science (Engel et al., 2016), a turn that follows a consensus among such thinkers that the general flaw of classical theories is their endorsement of a reductionist view of perception. James (1900), for instance, famously argued that the qualities we consider essential in perceiving a thing are those that are more practically important. This is because, for him, perception is not determined exclusively by the world but is rather a product of the permanent subject-object interplay in an action context. Similarly, for phenomenology, Merleau-Ponty (1942/1963) contended that perception is inextricably tied to human intentions and actions, therefore highlighting its pragmatic, situated and enactive nature. Following Hegelian insight, he asserts that perception never takes place outside the context of human *work* (Merleau-Ponty, 1942/1963, p. 162), the ensemble of activities by which individuals transform the world, bringing about, at the same time, new cycles of behaviour. Given that perception and action have a common functional value (i.e., knowledge and modification of reality), they should be considered two sides of the same coin. James Gibson (1979/2014), influenced by both pragmatism and Merleau-Ponty’s phenomenology, gathered these ideas under the title of *affordances*, proposing that we perceive things in terms of what we can do with them—what they afford. In this connection, any inquiry into perception that neglects its practical value is doomed to be meaningless (Babska, 1965). Noë (2004) also defends this idea from an enactivist perspective when saying that “perception is not something that happens to us, or in us. It is something we do” (p. 1). Similarly, Rietveld and Kiverstein (2014) proposed that the perception of the environment as resourceful hinges on a *situated normativity* related to our skills and the specific sociocultural contexts and material settings where we act.

If perception is the counterpart of action, then concepts can be seen as instruments to achieve ends (Sakharov, 1990). For example, Barsalou (1983, 1991) argued that understanding conceptualisation requires an examination of the nexus between categories and the goals these categories allow individuals to attain. Introducing a pragmatic turn on the debate, he argued that categorisation could hardly be understood without considering its instrumental value. In everyday life, categories such as food-i-should-eat-so-as-not-to-break-my-diet or things-i-should-bring-to-the-office are fundamental for activity planning. Because of their goal-derived nature, Barsalou called these *ad hoc categories*. An exciting aspect of these categories is that the elements they bring together are not necessarily perceptually similar. A packet of flour, an egg, a butter stick, a glass of milk and a bottle of vanilla essence do not share physical attributes, such as colour, weight, shape, or texture. What brings them together in the ingredients-for-a-cake category is their potential contribution towards baking a delicious vanilla sponge cake. From this standpoint, the potential that exemplars have to serve a common goal—referred to as *ideals* by Barsalou (1985)—emerges as a crucial determinant of the graded structure of a category.

### Cognition Is not Just About Materiality but Also With It

So far, following pragmatic approaches, we have suggested that mainstream concept theories have overlooked how fundamental action and its pragmatic contexts are for perception and conceptual thinking. A further issue with these theories—perhaps more severe—lies in their failure to recognize the cognitive relevance of material culture. Likely, this disregard stems from Descartes’ mind-body dualism, which continues to profoundly influence the cognitive sciences (Lowe, 2009; Wynn 2017). In general, (neuro)cognitive research assumes, like Descartes, that there is a qualitative difference between mental and material phenomena, and that “mind takes place in the brain” (Hünefeldt, 2018, p. 111). Although not all cognitive theories fully adhere to Descartes’ radical rationalist view, those who hold the default position of a representational theory of mind cannot but agree that “a cognitive agent’s mental states are symbols or structures, internal to the agent, representing the agent's world” (Maloney, 1986, p. 131). Conceptual reasoning is thus confined to the dimension of the *res cogitans*—the abstract mind. In these perspectives, at best, the material world plays a limited role: things are *referential occasions* for our conceptual system, only providing the sensory content it needs to develop and operate. For even when concepts refer to the things of the world, they are seen as skull-bound mental phenomena: their formation, manipulation, enrichment, and application occur in the mental realm, inside the head.

The reification of mind has also influenced empirical research about conceptual thinking. For example, many studies on conceptual development have employed—for the sake of experimental control—abstract and artificial stimuli whose structure is not analogous to real-world stimuli (Alessandroni & Rodríguez, 2020; Berger, 1997), as if the materiality of the world simply did not matter. Most psychological experiments are not carried out in the real contexts of everyday life, but in abstract and oversimplified ones. In these abstract experimental situations, detailed characteristics typical of the real environment—including not just the materiality of things, but also uncertainty, complexity, the instability of phenomena, dynamic and interactive social exchanges, and so on—are often entirely absent (Viale, 2024). This research attitude illustrates that the cognitive ecologies in which humans live are not thought to be particularly relevant to the design of experiments. For example, researchers often use drawings, images, computer screens, and replica objects to investigate the existence of concepts of the objects they represent. The conflation of objects with their representations in other semiotic systems adds notable difficulties to the research, as there is evidence that they are not cognitively equivalent entities (e.g., Arterberry & Bornstein, 2012; DeLoache, 2000). In other words, *things* cannot be interchanged for their representations without cognitive consequences. The fact that these problems are rarely discussed in the psychological literature (Alessandroni & Rodríguez, 2020) ratifies the marginal place that the material world has for cognitive inquiries.

To what extent is a sharp division between concepts and materiality reasonable? In *Experience and Nature*, Dewey (1929) noted that:

The notion that the universe is split into two separate and disconnected realms of existence, one psychical and the other physical, and then that these two realms of being, in spite of their total disjunction, specifically and minutely correspond to each other, presents the acme of incredibility (pp. 267–268).

For the pragmatist philosopher, dualisms lead to argumentative impasses, because they artificially divide the world and then try to overcome the gaps they create through theoretical contrivances, appealing to haphazard causative powers.

Contrary to those who exiled materiality from debates about human cognition, other authors have contended that the link between conceptual thinking and materiality is indispensable, and even that “concepts and conceptual change are as much features of artefacts and the external world as they are of thinking” (Säljö, 2018, p. 113). This standpoint can be traced to works by Galpérine (1966), who argued that concepts derive from the actions subjects undertake with real things, within cultural activities. Also, Podolsky stated that only by incorporating materiality into discussions about concepts would it be possible to understand their development as a concrete process, that is, as it happens in real life (Gulmans et al., 1995). This is because conceptual thinking develops whenever real subjects run into a real need for new general ways of thinking, in real life situations that involve the active engagement with real things. To better understand this pragmatic and situated proposal for the study of conceptual thinking, some concrete examples are in order.

While discussing how humans developed numerical thinking, Malafouris (2010) considers the case of the Near Eastern system of counting and the influence that the use of three different forms of materiality (i.e., clay tokens, envelopes, and pictographic tablets) might have had. He argues that it was the use of clay tokens for concrete counting that ultimately provided the material scaffolding necessary for the subsequent abstraction and conceptualisation of number. According to him, the use of three-dimensional clay tokens as enactive signs provided the foundations for the posterior use of two-dimensional markings on the envelopes to indexically refer to the tokens contained in them. These markings, in turn, scaffolded the iconic tracing of images of clay tokens on pictographic tablets and, finally, the use of symbolic numerals. In his view, “the clay tokens bring forth the numbers and make visible and tangible the manipulation of their properties” (Malafouris, 2010, p. 40). The concept of number is not thought of, thus, as the result of internal processes, but as emerging from instances of material engagement where the brain, the body, and the stable material structure of clay tokens converge (see also Overmann, 2017, 2019a).

Empirical evidence from developmental psychology also supports this explanation. For instance, Scheuer and Sinclair (2009) reported that the “one-to-one correspondence in spontaneous action or play, such as putting one stick in each cup, precedes correct counting by years” (p. 20). They argue that categories related to number emerge from *numerical explorations* in which children engage when acting in and responding to their rich environment. In that way, the numerical understanding of the world is grounded in sociomaterial practices such as distributing things, performing pointing gestures, pretend play, and object manipulation. Similarly, Alessandroni and Rodríguez (2017) argued, versus the Conceptual Metaphor Theory (Lakoff & Johnson, 1999), that the development of the containment concept is not the natural by-product of processes of abstraction from embodied experiences, but is embedded in the actual use of various objects *as containers* in the context of specific pragmatic challenges. As an illustration, in the 0–1 and 1–2-year-old classrooms of the infant school babies regularly participate in the *treasure basket* and *heuristic play* activities where teachers guide them in performing properly the put in/take out, fill/empty, open/close, and cover/uncover actions with different containers. The establishment of the containment function of objects is, in that context, an educative purpose and a developmental achievement. In other words, things are not *a priori* containers, but *become* containers by means of practice in material engagement situations (for a long-term archaeological perspective on the issue of containment, see Knappett et al., 2010).

## MET: Cognition as Material Engagement

In previous sections, we outlined a perspective on conceptual thinking that thoroughly considers (i) the situated and enactive nature of perception and (ii) the cognitive role of material culture in the everyday activity contexts where categorisation takes place.^3^3To illustrate, see Goodwin’s perspective on colour categorisation within his practice-based theory of knowledge and action (Goodwin, 1994). Research in the cognitive sciences is in great need of this perspective because, until now, action is largely missing from the table, and materiality is usually reduced to the role of the *mise en scène* of cognition. In the last section of this paper, we propose a novel perspective on conceptual thinking, based on the Material Engagement Theory (MET) (Malafouris, 2004, 2008a, 2013, 2019, 2020), a cross-disciplinary analytical framework grounded in the anthropological archaeology of mind, aimed at investigating how people and things co-constitute each other. With an eye to providing the right context for our main proposal, we first summarise the three working hypotheses of MET. The *extended mind hypothesis* asserts that cognitive processes cannot be studied without paying attention to the co-constitution relations between brain, body, and world. The *hypothesis of enactive signification* proposes that material culture has unique semiotic characteristics that give rise to processes of signification that follow an enactive logic of sense-making. The *hypothesis of material agency* defines agency as the product of situated activities where materiality has an active role.

### The Mind Is Extended

When it comes to determining the whereabouts of cognition, the classic answer is that cognition happens in the head, which delineates the boundaries of a mental space where computations over representations take place. However, myriad non-Cartesian theories have called into question the traditional approach, giving rise to 4E perspectives (i.e., embodied, embedded, enactive, and extended) (for a recent discussion, see Gallagher, 2017, 2023). Aligned with these approaches, MET posits that human cognition is extended, inasmuch as it cannot be circumscribed in a single place (e.g., the brain). It is an embodied and environmentally embedded phenomenon, with an agent’s mind and cognitive processes incorporating resources and processes from the physical world as integral components (Malafouris, 2013, 2018; see Clark, 2008). The extended point of view is particularly useful when accounting for real-life situations where perceiving, thinking, doing, and making are co-defined in such a way that they are inseparable from each other (Malafouris, 2019). That is why, for MET, studying the mind involves examining how, in concrete situations, brain, body, and culture *conflate*^4^4For MET, "brain", "body" and "culture" are analytical abstractions that can only exist interdependently through processes of material engagement. As a result, establishing conceptual boundaries between these three terms is considered unhelpful. Rather, MET speaks of these three terms as *conflated*, that is, fused together. (Malafouris, 2004), thus configuring the relationships between the mental, social, material, and bodily processes that make up our *cognitive ecologies* (Hutchins, 2010).

Various empirical studies have shown the usefulness of studying cognition from extended and situated viewpoints. For example, Kirsh and Maglio (1994) provided evidence that, during a Tetris game, certain translations and rotations can work by relieving the cognitive load of problem-solving processes intrinsic to the game, even if they are not intended to improve board position. The central idea is that these apparently fortuitous epistemic actions are indispensable for controlling behaviour in real-time situations that demand split-second decisions. Similarly, there is evidence that the use of manipulatives, gestures, and body movements, more than the use of abstract symbols, supports mathematical thinking (e.g., Carbonneau et al., 2013; Overmann, 2017, 2019a), and that activities such as drawing, sketching, doodling (e.g., Glaser, 2008), and handwriting (e.g., Menary, 2007) reorganise our thought processes, making them smoother and more flexible. This resonates well with Goodwin’s claim that the articulation of graphic representations (e.g., the production of maps by archaeologists) and the engagement with professional tools (e.g., Munsell colour charts^5^5A Munsell colour chart is an ordered grid of colour samples featuring circular holes that allow the colour of the samples to be compared with the colour of other materials (e.g., a sample of dirt on a trowel).) restructure perception and bring about new ways of organising scientific knowledge (Goodwin, 1994, 2002). On a similar note, Malafouris and Koukouti (2018) argued that individuals’ recall of practical activities (e.g., riding a bicycle) is inherently tied to their (re-)enactment within specific sociomaterial contexts. In this view, memory is an interactive and transactional process, an active entanglement of neural, bodily, and material resources. Similar arguments have been advanced in the case of material imagination (Koukouti & Malafouris, 2020) and creativity (Malafouris, 2014; Poulsgaard, 2019; Poulsgaard & Malafouris, 2023, 2017; Vallée‐Tourangeau, & March, 2020). Finally, it is worth noting that extended approaches have consequences for the study of development, since, for instance, it would not be possible to have an abstract concept of weight without first experiencing “the sense of weight that we encounter through the pull on our muscles as we lift objects” (Tall, 2008, p. 5; see also Renfrew, 2007).

### Materiality Is Meaningful

While endorsing an extended perspective, MET differs from other extended approaches that, by relying on the *multiple realisability causal model* (de Jong, 2003), assume that mental states are *functional states* that can be equally carried out by different material systems (e.g., the well-known example of Inga and Otto, as discussed in Clark & Chalmers, 1998). An important difficulty with functionalism is that it neglects the inquiry into the particularities of the objects that “instantiate” the mental states in each case (i.e., objects become *contingent*) (Farkas, 2012). Against functionalism, MET advocates for the *primacy of material engagement*. In other words, as we engage with the world, materiality envelops our everyday thinking and experience, thus mediating and *constituting* our ways of being and developing in the world (Malafouris, 2008a). Mental states are never independent of their physical realisation—matter matters. Materiality constrains and enables our cognitive processes, so that things become meaningful in regard to what they are and what we do with them. This is a sense-making process that Malafouris (2013) deems *enactive signification*.

However, *material signs* (see Iliopoulos & Malafouris, 2021) do not work like linguistic signs which, in the language-as-a-code traditional perspective, are seen to refer arbitrarily to a meaning that exists independently of the signs themselves (Love, 2004). Material signs “can be touched, carried, worn, possessed, exchanged, stored, transfigured, or destroyed” (Malafouris, 2013, p. 95). These enactive properties, which comprise the *enactive semiotic idiom* of things, make the classical priority of the signified over the signifier untenable (Malafouris, 2008b). Much as in language use where “the how of the saying influences not only the way in which our listeners come to understand us as speakers but also what they hear us saying” (Gahrn-Andersen, 2019, p. 177), the how of the engaging with things (what Merleau-Ponty calls an *embodied style*) shapes how signifier and signified originate from sociomaterial practices. In this way, material signs *participate* in the meaning that is inscribed in them while conveying it (Malafouris, 2015, 2016).

### Agency in Material Engagement

MET also claims that materiality contributes to agency in a primary sense. This view is fundamental, as it dissolves another long-standing assumption: the *agent-things dualism*. Classically, agency is seen as a capacity of individuals to act intentionally, and things are means for externalising previously formed intentional mental states. For MET, in contrast, the agent is not *a priori*, but emerges *in* and *thanks to* processes of material engagement—an agent is not such before its encounter with materiality. Agency is a relational property: a property of material engagement rather than a property of the actor *simpliciter* or the thing *simpliciter* (Malafouris, 2008a, p. 22). It refers to the continuous product of the transactional *push-and-pull relationships* between an organism and its world which bring about new modes of acting and thinking (Ransom, 2019). In harmony with this idea, MET states that thinking is better characterised as *thinging*, something we do *with* and *through* things rather than simply being *about* them (Malafouris, 2020).

This take on agency and thinking makes MET a much more comprehensive paradigm than many so-called “weak embodied” perspectives (Alsmith & de Vignemont, 2012) that provide only a “shift from the disembodied computational image of mind to one that is now grounded in neural structures and brain networks” (Malafouris, 2016, p. 291; see also Booth, 2016; Turner, 2011). Whereas such weakly embodied views highlight the importance of brain activity and bodily experiences for cognition, they neglect the study of embodied action as it occurs within the situated and dynamic interactions with material culture that brings forth the world and its meaning. The *act of embodying* (Malafouris, 2013, 2016), as MET understands it, comprises a series of transactions whereby “our body, as a unit of meaning, participates in the co-construction of cognitive processes in specific material and cultural contexts” (Alessandroni, 2018, p. 243), bringing together brains, bodies, and the material culture.

Plastic changes in the brain cannot be split off from other forms of plasticity occurring in the broader sociocultural context where action takes place. Thanks to *metaplasticity* (Malafouris 2010)—the constitutive intertwining between neural and cultural plasticity—“material culture competes equally with any other brain or behavioural region for a place in the non-genetic heritable structure of the human cognitive system” (Malafouris, 2015, p. 360, see also Bruineberg & Rietveld, 2019; Kiverstein & Miller, 2015). In a related vein, Ryan and Gallagher (2020) argued that the brain could be better described as a *resonant organ* rather than a representational one, which has significant consequences for cognitive studies: “since [if] we can actively construct or reorganise an environment to enhance resonance processes, or to make the environment resonate with us, [then] a full account of resonance must explain this part of the process as well” (Ryan & Gallagher, 2020, p. 10; see also Gibson 1966/1968, 1979/2014 and Raja, 2018 for related applications of the notion of *resonance*). Neurocognitive research has endorsed these relational perspectives on brain activity. For example, there is now evidence suggesting that the power of an object to trigger an action depends upon the effective possibility that an individual has to interact with it (Costantini et al., 2010), that goal-directed interactions with objects can affect action processing in the brain (e.g., object categorisation) (Triberti et al., 2016), and that “what people are doing may impact on their perceptual judgments on the surrounding things” (Costantini et al., 2019, p. 1805).

## Conceptual Thinking as Conceptual Thinging

In the preceding sections, we have provided an overview of the fundamentals of MET. However, questions persist about the link between MET and conceptual thinking. How do extended mind, enactive signification, and material agency relate to conceptual thinking within MET? In what sense would materiality be constitutive of conceptual thinking?

According to MET, a concept could never be said to inhabit the brain: conceptual thinking—like any other form of cognition—is relational and found within sociomaterial practices where humans carry out meaning-making and sign-construction processes. This clearly contrasts to a weak embodied view. For example, Barsalou (2017) proposed that “a concept is a dynamical distributed network *in the brain* coupled with a category in the environment or experience” (p. 10, emphasis ours), and Pexman (2019) defined concepts as knowledge representations that “correspond to patterns of activation *across numerous neural areas*” (p. 1274, emphasis ours). Whereas these and other weakly embodied views highlight, at best, the importance of body-related brain activity for cognition, they neglect the study of embodied action as it occurs within the situated and dynamic interactions with material culture that brings forth the world and its meaning.

To define conceptual thinking, we start from the epistemological foundations of MET: cognition is a transactional process that occurs in and thanks to our material engagement with the world. Brains, bodies, and the world all contribute to the construction of meanings. From this point of view, one can only study people and things “as worldly, not just as in the world, but as incorporated into practices in the world” (Costall, 2012, p. 93). Consequently, we advance our first principle:

P1: Conceptual thinking is a specific form of material engagement that individuals become able to sustain with their environment.^6^6Note that we are not arguing that so-called “abstract concepts” (e.g., the love concept) do not exist. As is the case with other “abstract” cognitive phenomena (e.g., imagination or memory), it is very likely that “abstract” concepts are more material than is usually recognised (i.e., that abstract conceptual thinking involves actual material engagement) (Malafouris, 2020). It should also be pointed out that abstract conceptual thinking is not the starting point of our cognitive system, but a developmental achievement that supervenes on minimal ways of conceptually engaging with material culture.

Clearly, not every case of material engagement can be called conceptual. To qualify as such, it must result in generalisation, which entails interacting with multiple category exemplars rather than individual entities. From MET’s semiotic approach, the generality of conceptual thinking cannot be reduced to the aggregation of similar sensory impressions “emanating” from things, as is the case in traditional theories. This is because things are much more than sensory collections. Given that things are embedded in structured material settings (Rietveld & Kiverstein, 2014) and networks of culturally evolved social practices and routines (Le Groupe µ et al., 2013; Rączaszek-Leonardi et al., 2019), they are endowed with semiotic properties (Malafouris, 2016) that transcend inherent properties (e.g., shape or weight). As a result, in the *thinging* process individuals engage not only with the physical properties of objects but also generate meanings that, upon re-enactment, generalise and, as we will argue, lead to concept formation.

An excellent example of a semiotic property of objects concerns knowledge about *object functions*. *Conventional object uses* (e.g., cups should be used for drinking) are culturally preferred forms of engaging with things. As such, they cannot be reduced to the “physical” properties of objects (Knappett, 2010, see also the notion of *canonical affordances* in Costall & Richards, 2013). To become skilful tool users, children first need to grasp the cultural norms of object use (Rodríguez, 2012). Several studies have shown that babies begin to use artifacts according to their cultural function around their first birthday (see Rodríguez et al., 2018). And since object functions only become available when demonstrated (Horst et al., 2005, p. 616), this cognitive achievement depends upon communication processes that take place through a wide range of semiotic resources (e.g., *use demonstrations; ostensive, indexical, and symbolic gestures; language*) that adults deploy within situations of triadic interaction (adult-baby-object), specifically in contexts of “natural pedagogy” (Alessandroni, 2023; Gergely & Csibra, 2013; Nonaka & Goldfield, 2018; Nonaka & Stoffregen, 2020; Rodríguez & Moro, 1999).

Considering the semiotic complexity of the material world, rather than conceptual thinking taking its general character from the gathering of similar things at the physical level, or from the gathering of abstractions in some kind of purely mental realm, we propose that:

P2: Conceptual thinking (i.e., conceptual material engagement) is general inasmuch as it aggregates common, recurrent enactive meanings.

When we speak of meaning, we are not referring to the semantic content of mental representations, but to the “emergent product of the relational process of engagement with the material world” (Iliopoulos, 2016, p. 114). In this performative sense, *meaning* refers to how individuals create, discover, perceive, and act upon affordances, which are the possibilities for interaction that specific material engagement processes bring forth. *Meaning-making,* in turn, is the process of creative enrichment of the transactional resourcefulness of the environment that material engagement situations spur. Indeed, every instance of material engagement opens up new opportunities for interaction, scaffolds further semiotic encounters with the world (Hoffmeyer, 2014), and sets up *action boundaries* that constrain subsequent engagement. Further, as certain forms of material engagement are more relevant than others within specific sociomaterial practices, they become recurrent. For example, in specific contexts—usually modern and Western based—while eating at the table, the most relevant way of engaging with spoons, forks, and knives is, typically, to perform conventional uses. This recurrence over time gives rise to general enactive (i.e., action-oriented) meanings and fosters the construction of *potentials for behaviour* that can be transferred “across extended periods of time and across different environments and situations” (Adolph, 2019, p. 182). To use the same example, throughout life, individuals become skilled at engaging with various eating artefacts belonging to the same class (e.g., spoons) in similar functional ways. Asserting that material engagement can have a general nature acknowledges the fact that individuals come to engage with specific environmental affordances in recurrent meaningful ways.^7^7See Bruineberg et al. (2019) for a discussion on *general ecological information* and attracting affordances (*solicitations*), and Heras-Escribano et al. (2024) for a related analysis of embodied concepts as affordance-based states bridging experience and discursive or explicit conceptual content.

Wertsch (1998) supports this idea when noting that “once one becomes somewhat proficient at riding one bicycle, one is not limited to riding that bicycle alone” (pp. 31–32, see also Oakes, 2008, p. 255). Indeed, thanks to the real-time entanglement with materiality, subjects become more and more skilful in knowing how to turn the material world into an active resource (Malafouris & Koukouti, 2018, pp. 174–175). Through material engagement, individuals develop the ability to generalise forms of know-how and apply them in future interactions. We argue that expanding functional behaviours to new exemplars within a category in line with shared socio-cultural norms of object use is tantamount to developing a kind of conceptual thinking (i.e., conceptual material engagement). What unites the various instances of practical thinking within this engagement is not perceptual similarity, but functional resemblances. These functional resemblances minimise uncertainty about how to engage with the environment through skilled agency, provide new foundations for successful interaction, and allow the subject to move from an undifferentiated environment to a situation where some particular affordances are more relevant than others (de Carvalho & Rolla, 2020). As Brinck and Reddy (2020) put it, “increasing material engagement results in new ways of organizing actions and groups with new concepts developing that reflect the changes, and interaction with these concepts generates feedback-loops that cause re-organisation of the actions and groups, et cetera” (p. 34).

As a result, successful material engagement brings forth new degrees of *semiotic freedom* (Hoffmeyer, 2015), the capacity to derive useful information by help of semiosis. This takes us to our third principle:

P3: Conceptual thinking brings forth new types of semiotic constraints (i.e., changes in the semiotic landscape of affordances), allowing subjects to perceive and use different types of meanings in increasingly varied contexts.

As per MET, materiality could not but be active in these processes of signification. Things are fundamental because they act as *material anchors for conceptual blends**^8^*8According to Fauconnier and Turner (2002), *conceptual blends* are integration networks where the projection and elaboration of two input mental spaces lead to the construction of a novel blended space with emergent dynamics. Hutchins' studies have convincingly shown that input spaces need not be representational or abstract: material culture can provide direct input to conceptual blending processes. (Hutchins, 2005) and support concept formation by “enhancing and tightening conceptual blends in a memorable and durable manner” (Malafouris, 2013, p. 104). In everyday life, “material elements are taken as proxies for relationships among conceptual elements” (Hutchins, 2005, p. 1562). In this regard, concepts are very much material. In recent works, Overmann (2019b, 2023) exemplified this showing how the concept number acquired, throughout history, different structural properties as new material forms were incorporated into the cognitive system for numbers (i.e., fingers, tallies, tokens, and notations). In effect, putting such *things* to use constitutes conceptual formation and reformation; without processes of material engagement, neither cognitive processes nor, therefore, concepts could exist. Extending Malafouris’ notion of human cognition as a *hylonoetic*^9^9The term *hylonoetic* comes from the Greek *hyle* (matter) and *nous* (mind). Malafouris (2013, p. 236) advocates a *hylonoetic ontology* (i.e., centred on the processes of thinking *through* and *with* matter) opposed to the orthodox *hylomorphic ontology,* which assumes thinking to be the imposition of form (Greek *morphé*) *on* matter.
*field*—a mindscape extending into the extra organismic environment (Malafouris, 2013, 2019)—we suggest that:

P4: Conceptual thinking always takes place within a hylonoetic field constituted by bodily practices and artefacts, often in the presence of and in interaction with others.

During ontogenesis, action develops “not as an individual act, but as a social one: by exchanging things with the Other, by touching things, and looking at them with the Other” (Werner & Kaplan, 1963, pp. 42–43). In other words, most actions that children come to perform with things are, in the beginning, collective projects embedded in a community of practice (Lave & Wenger, 1991/2008; Vygotsky, 1934/2008). There is empirical evidence that triadic interactions involving material culture (adult-object-baby) occur from the beginning of life and have a significant influence on the shaping of cognitive processes (e.g., Alessandroni et al., 2020; Mendoza-García & Moreno-Núñez, 2023). This is so because “even when the child cannot blend in the same communicative act one object and another person, he is *placed by others in meaning-loaded material scenarios*” (Alessandroni et al., 2020, p. 1556, emphasis ours). Caregivers act as ambassadors for their children, dynamically modulating the opportunities for action their infant can access (Moreno-Núñez et al., 2017; Nonaka & Goldfield, 2018). Simultaneously, caregivers introduce their infants to the culturally relevant action formats that adults already embody during interaction (Kärtner, 2018). As Gallagher (2020b) nicely puts it, “other people afford a variety of interactions” (p. 10) and, in doing so, they (en)actively shape the ways children interact with things. Besides granting greater semiotic freedom, conceptual thinking in material engagement enhances communication among individuals, thus gaining social relevance throughout life. For Gallagher and Ransom (2016), for example, across the life span, material culture is fundamental for social coordination: joint action is “materially constrained and materially enabled” (p. 349), and material artefacts allow for the coordination of social forces in unique ways.

Without doubt, recurrent patterns of activity involve a non-discursive, enactive, and situated normativity: once specific interactive formats become ritualised (Rączaszek-Leonardi et al., 2019), adults expect children to be increasingly capable of engaging with material culture in a skilful and stable manner. Thereby, action becomes “subject to normative assessment as better or worse, as more or less correct given the specific demands of the situation” (Kiverstein & Rietveld, 2018, p. 154). The points we have made so far bring us closer to a fuller understanding of the complexity of the *circumstances of action* (Gallagher, 2020b), which involve not only elements such as the movements of the agent in her environment, her skills, her intentions, her interactions with others, the objects/artefacts available in the environment, their physical and sociocultural characteristics, and the effects or consequences of the action, but also how other people, as interpreters of the action, operate (see Gallagher, 2020b, p. 12).

To summarise, the point we are making here is that conceptual thinking in material engagement is intersubjectively enabled and constituted. Paraphrasing Cole (1985, p. 158), we would say that conceptual thinking is *peopled by others.* Due to these essential linkages between conceptual material engagement and socialisation, we propose that:

P5: Conceptual thinking always originates in the cognitive context of sociomaterial normative practices.

Finally, it remains to address the problem of conceptual codification: What could concepts be if not propositional representations? According to MET, the effectiveness of material culture does not consist in its potentiality to be represented, but in the fact that it allows us to think and gain new understanding by engaging with it (Malafouris 2013, 2016). In other words, thinking as “thinging should not be understood as a psychological process of internalisation or representation” (Malafouris, 2019, p. 7), but as an action-oriented engagement. Focusing on the actual processes of organism-environment interaction, we are arguing that conceptual thinking should be investigated as a case of skilled performance that takes place in material engagement.

Our view aligns with contemporary contributions that do not simplify knowledge to the possession of well-supported beliefs, nor reduce rationality to the contemplation of rules in the mind (e.g., Hetherington, 2011; Melser, 2004; Schwitzgebel, 2013). According to such critical epistemologies, intelligent action is a practice or custom in which an agent sustains a proficient interaction with the environment, which requires the coordination of cognitive abilities to situational constraints (e.g., Rolla, 2021; see also Wittgenstein, 1953/2009). Therefore, it is plausible to consider concepts as *enactive dynamics* of a general nature that unfold *in* and *with* the world. Similar to the term “mind” (Malafouris, 2019, p. 5), the term “concept” can be better understood as a verb:

P6: We *concept* every time we proficiently take part in material engagement processes through concept-like behaviours.

Examples of these behaviours are to engage in a similar way with different members of a category (e.g., to drink from several cups), and to establish differential engagement processes with members of two or more different categories. These kinds of behaviours can be observed from very early ages at the infant school, where children as young as 11 months of age already materially engage in a conceptual way with several things (e.g., pacifiers, cups, and bibs). Besides, as children develop, they become more skilled at bringing forth canonical ways of materially engaging with the world to fulfil essential functions such as self-regulation, communication with others, and understanding others’ intentions (see Alessandroni, 2021, 2023).

It is worth emphasising that we are not just claiming that conceptual thinking develops thanks to our engagement with material culture (i.e., that materiality plays a causal role for conceptual thinking), but also the stronger hypothesis that certain instances of material engagement are actual cases of conceptual thinking (i.e., that materiality plays a constitutive role for conceptual thinking). If concepting is not a matter of forming mental representations but of practically engaging with the material and social world, then conceptual thinking is to be found distributed within the cognitive system in which such engagement develops, and not in a subsystem of one of the system’s participants (i.e., the individual’s cognitive system). Malafouris (2013) sums it up by saying that “there is no mind behind the artefact” (p. 34). In other words, concepts inhabit the practical contexts in which they emerge:

P7: A concept is practically instantiated in the brain-body-world enactive network from which it emerges thanks to processes of material engagement.

The consideration of these principles (P1–P7) leads us to an ecological-enactive, pragmatic, distributed, and semiotic definition of conceptual thinking:

Conceptual thinking is a case of unmediated practical knowledge individuals put into play every time they engage, in a generalising manner, with and through the world. Developmentally, concepts can be described as *semiotic bundles* of material engagement processes: common meanings built through our history of material engagement. As such, they come down to the dynamic coordination of bodily practices and artefacts that takes place in the arena of sociomaterial activities. In short, to “have a concept” is to be able to think-in-action, with real things, enacting and re-enacting the same pragmatic principles with different exemplars of the same class. In that regard, we propose to understand “concept” as a verb—a way of participating in material engagement through concept-like behaviours. Since engaging with things contributes constitutively to conceptual thinking, conceptual thinking is also a kind of material skill, relationally dependent on things: without objects, there would be no concepts. That is why, in our view, concepts belong to subjects as much as they belong to things. Once developed, concepts act as potentials for behaviour. They operate as affordances (Gallagher, 2017), scaffolding further interactions with the world and allowing individuals to gain access to higher levels of semiotic freedom and to perceive and use different types of meanings in increasingly varied contexts. As concepts exemplify how materiality brings about new modes of acting and thinking, conceptual thinking is, in fact, *conceptual thinging*.

## Wrapping Up

In this article, we advocated for a significant shift in our understanding of conceptual thinking, utilising Material Engagement Theory (MET). We began by addressing two critical issues within mainstream concept theories. The first one involves the understanding of concepts as perceptual abstractions that gather things that look alike. Such a perspective fails to consider the pragmatic contexts that give meaning and structure to perception. The second problem is the oblivion of the cognitive relevance of materiality. In confining concepts to the dimension of the abstract mind, mainstream theories detach conceptual thinking from material culture: conceptual activity occurs in the mind, and things are epiphenomenal.

In contrast, empirical research has recently emphasised the superlative importance of material culture for cognition and argued that conceptual thinking cannot be defined without acknowledging our interactions with real things within cultural activities. In this context, we argued that MET offers an adequate framework for reconsidering the nature of cognition. For MET, cognition happens in the dynamical relations between brains, bodies, and the world, thanks to processes of material engagement that give rise to enactive meanings. Cognition is situated within cultural activities where materiality plays an active role. In this respect, we advanced seven principles for redefining conceptual thinking. According to MET, conceptual thinking is a form of unmediated practical knowledge enacted whenever individuals engage with materiality in a generalising manner. It originates from recurrent sociomaterial practices and involves the construction of common enactive meanings and action boundaries through *thinging* (i.e., thought processes with/through/about things). A concept is not a definition codified in the mind but an act of participation in material engagement processes. In that respect, conceptual thinking is distributed across the brain-body-world enactive network from which it emerges.

By focusing on the dynamics of our interaction with the world and the constitutive role of material culture for conceptual thinking, our proposal counters orthodox representational and abstract perspectives, addressing the issues outlined in the first section of the paper. Furthermore, our sociomaterial practice-based framework provides a robust foundation for empirical research, facilitating a deeper understanding of the cognitive ecologies where conceptual material engagement takes place.^10^10For a detailed discussion about contemporary issues and challenges of empirical research on conceptual thinking in developmental psychology, see Alessandroni and Rodríguez (2020). This perspective contributes to the much-needed *material turn* in the sciences of the mind, offering insights into how materiality makes us who we are.
